# The Importance of Differential Diagnosis in Splenogonadal Fusion: A Case Report

**DOI:** 10.7759/cureus.54454

**Published:** 2024-02-19

**Authors:** Inês Coelho Mogárrio, Carla Pilar, Ema Santos, Fátima Alves

**Affiliations:** 1 Pediatric Surgery, Hospital Central do Funchal, Funchal, PRT

**Keywords:** differential diagnoses, orchidectomy, congenital anomaly, testicular mass, splenogonadal fusion

## Abstract

Splenogonadal fusion is a rare, benign congenital malformation characterized by the association of splenic tissue and gonads (typically testicles). It is a condition of male predominance and can be classified into two types: continuous, if the spleen and gonad are united by a splenic cord or fibrous tissue, or discontinuous. Splenogonadal fusion is often associated with other congenital anomalies such as cryptorchidism, limb defects, and micrognathia. Differential diagnosis can be difficult and includes inguinal hernia, spermatic cord cyst, cryptorchidism, or testicular mass. Due to little knowledge of the pathology, unnecessary orchidectomies are often performed.

A previously healthy five-year-old boy was sent to a pediatric surgery appointment due to testicular asymmetry. The physical examination showed a painless, nodular mass adhering to the upper pole of the left testicle, without any palpable inguinal masses. Tumor markers were negative, and a testicular ultrasound with Doppler revealed a mass suggestive of an accessory testicle. Left inguinal surgical exploration revealed the presence of a mass joined by fibrous tissue to the upper pole of the testicle, but no connection to the native spleen was found. Total excision was performed with the testicle’s preservation. The anatomopathological analysis revealed morphological aspects compatible with splenic tissue with normal characteristics.

The diagnosis of splenogonadal fusion is rare and complex, requires several differential diagnoses, and is often made intraoperatively.The prognosis is excellent as long as there are no associated malformations. A high level of suspicion for this pathology, with recognition of the anatomical structures, can avoid unnecessary orchidectomy.

## Introduction

Splenogonadal fusion is a rare, benign congenital malformation characterized by the association of splenic tissue and gonads (typically testicles) [1-5]. The first case was described in 1883 by Bostroem, and the first review of the pathology was described by Putschar and Manion in 1956, who created a classification system [1,2,5].

Splenogonadal fusion is a condition of male predominance; however, the diagnosis in females is more difficult because the ovary is not visible on physical examination [3,4,6]. It can be classified into two types: continuous, if the spleen and gonad are united by a splenic cord or fibrous tissue, or discontinuous [3]. The discontinuous type consists of gonadal fusion with an accessory spleen or ectopic splenic tissue and is typically not associated with other congenital abnormalities [4,7]. Splenogonadal fusion is more common on the left side, and the continuous form is more prevalent [8].

Etiology is not completely understood, but it is thought that between the fifth and eighth weeks of intrauterine life, the proximity between the cells of the dorsal mesogastrium, which form the spleen, and the cells of the gonadal fold, promotes this fusion by direct contact and/or inflammatory changes [1-4,8]. During embryonic development, the descent of the testis could draw out the developing spleen fused to the testis [1].

Continuous form is often associated with other congenital anomalies such as cryptorchidism, limb defects, and micrognathia. Other associated congenital malformations, although rarer, are heart defects, cleft palate, anal stenosis, spinal bifida, Moebius syndrome, hypospadias, osteogenesis imperfecta, persistent Mullerian duct syndrome, Potter, syndrome, and gastrointestinal malrotation [1,3].

The clinical presentation is often asymptomatic, manifesting as a palpable testicular mass; however, in older children, it can cause pain [6].

Differential diagnosis can be difficult and includes inguinal hernia, spermatic cord cyst, cryptorchidism, or testicular mass [1,6]. Testicular ultrasound with Doppler has an important role in the diagnosis; however, the increase of vascularization present in splenogonadal fusion can mimic a testicular neoplasm [3].

This entity is often an incidental finding during exploration for scrotal mass, cryptorchidism, or inguinal hernia [1]. Due to little knowledge of the pathology, unnecessary orchidectomies are often performed [1,3,5], significantly impacting the patient's quality of life.

In this paper, we present a clinical case of discontinuous splenogonadal fusion, with testicular preservation.

## Case presentation

A previously healthy five-year-old boy was referred to a pediatric surgery appointment by his attending physician due to testicular asymmetry. The patient’s medical and family history revealed nothing unusual.

The patient denied any other symptoms, such as testicular pain, testicular swelling, or urinary symptoms. Physical examination showed an appropriate weight status for his age and good general health. Both testicles were present in the scrotum with adequate consistency. A painless nodular formation was palpable in the upper pole of the left testicle, exhibiting well-defined contours and adherence to the underlying planes. No palpable inguinal masses or other physical changes were found.

Blood tests, including lactate dehydrogenase, beta-human chorionic gonadotropin, and serum alpha-fetoprotein were all negative. Testicular Doppler ultrasound revealed a mass in the upper third of the left testicle with 1.5 cm in length, well-defined contours, and increased vascularity, suggestive of an accessory testicle.

**Figure 1:**
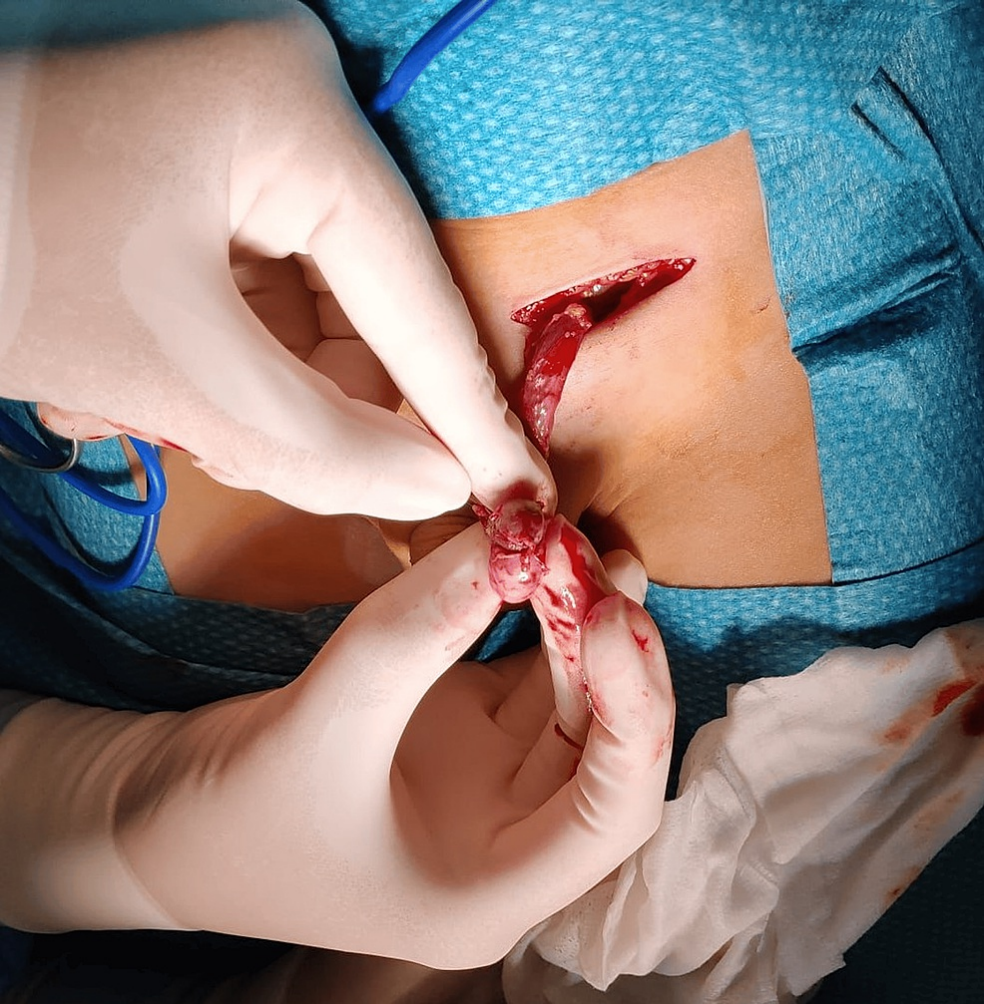
Discontinuous splenogonadal fusion. Note the absence of a cord connecting the gonad to the spleen and the presence of an accessory spleen anchored to the testis.

Left inguinal surgical exploration showed the presence of a mass joined by fibrous tissue to the upper pole of the testicle, but no connection to the native spleen was found (Figure 1).

**Figure 2:**
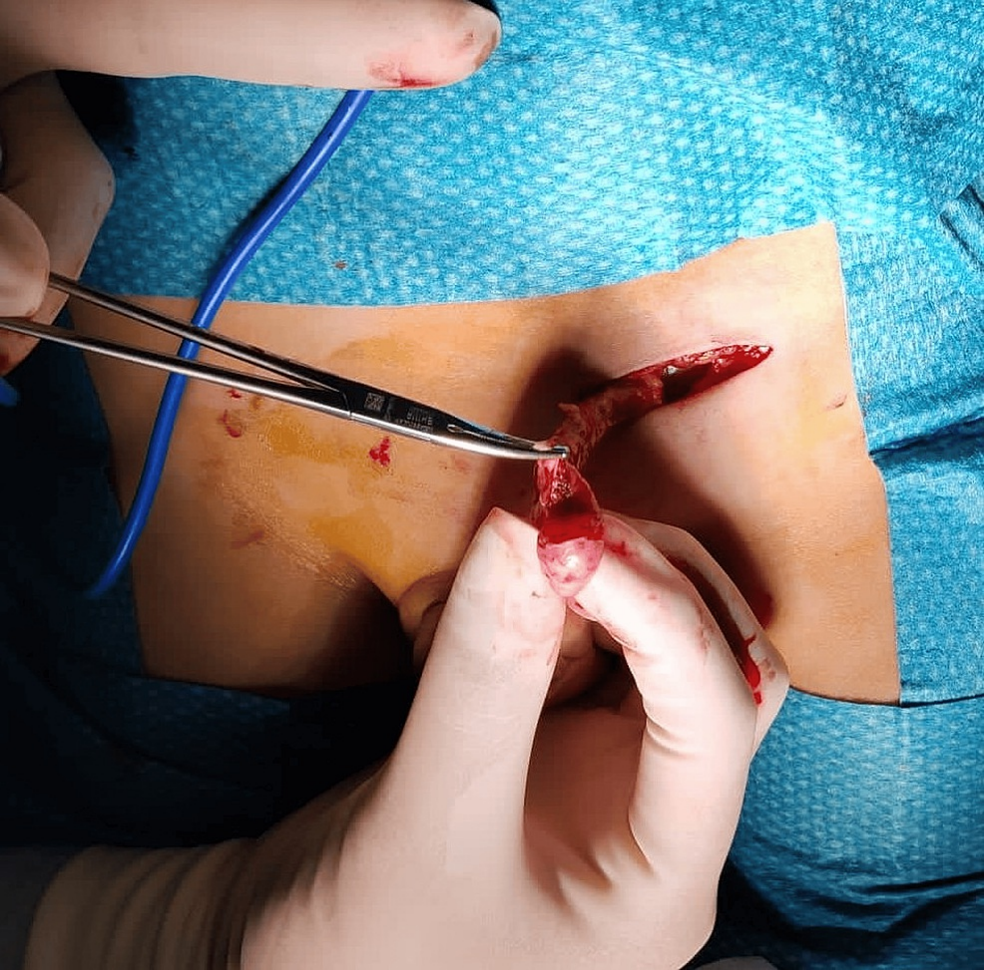
Excision of splenic tissue with preservation of the testicle and without damaging the tunica albuginea.

**Figure 3:**
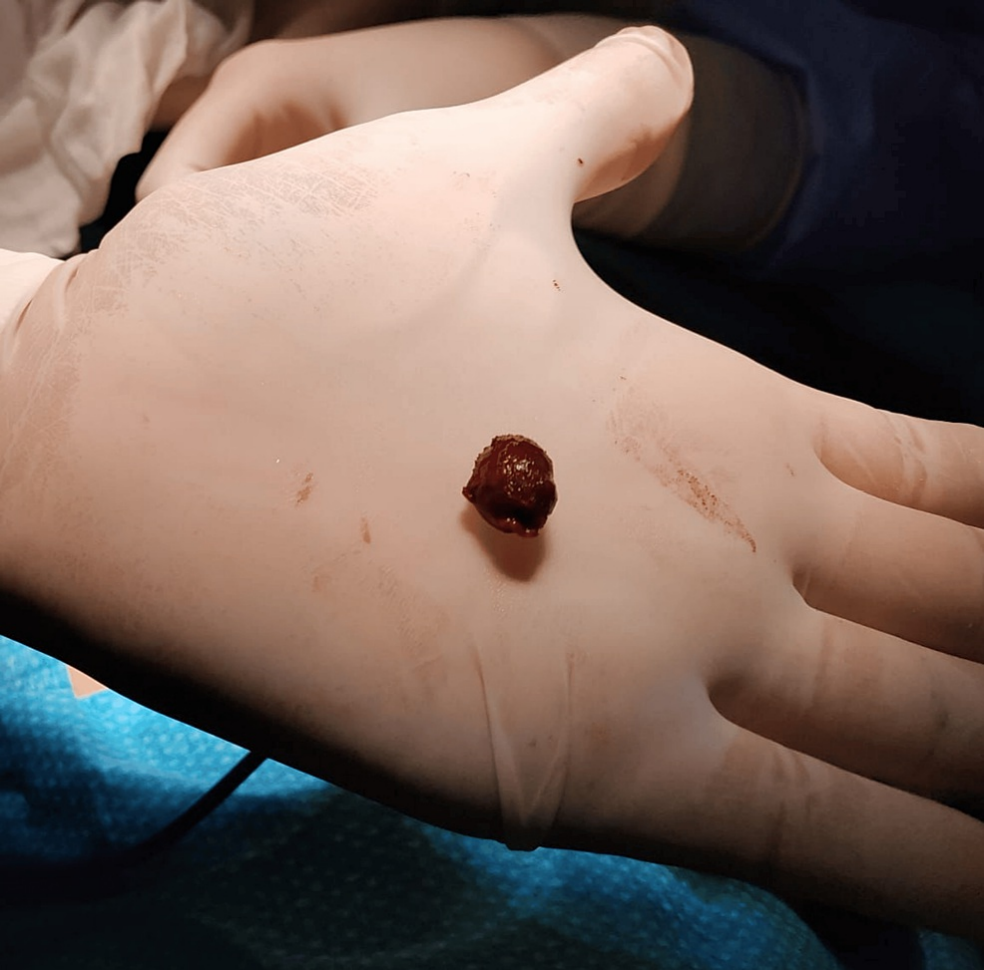
Ectopic spleen without any fibrous cord attached.

A total excision was performed without damaging the tunica albuginea, ensuring testicular preservation (Figures 2-3).

The postoperative period was uneventful. The anatomopathological analysis revealed morphological aspects compatible with splenic tissue with normal characteristics. The patient underwent an abdominal ultrasound in the second month after surgery, which did not reveal any other supernumerary spleens. Currently, with one year of follow-up, the patient is clinically well, with no complaints.

## Discussion

Splenogonadal fusion is a rare congenital entity, with only around 220 cases described in the literature [1]. The presented case aligns with the existing literature, involving a male patient with splenogonadal fusion on the left side. The increased occurrence on the left side is explained by the proximity between the spleen and the left gonad during embryogenesis [1].

Splenogonadal fusion can be classified as continuous or discontinuous, with the continuous form representing the majority of cases, around 55% [1]. In this case, we present the discontinuous form, in which an ectopic splenic tissue was directly attached to the gonad, without connection to the native spleen, and no associated malformation, which is in accordance with the literature [4]. In the continuous type, about 31% of splenogonadal fusion patients had cryptorchidism or inguinal hernia [1].

Our patient was a male child with a painless testicular mass, negative tumor markers, and a testicular Doppler ultrasound that suggested an accessory testis. On Doppler ultrasound, splenogonadal fusion may present as an encapsulated homogenous and hypoechoic lesion with internal vascularity; however, testis cancers can present identically [4]. Unable to exclude a testicular neoplasm, surgical exploration was carried out by inguinotomy.

Computed tomography and magnetic resonance imaging show similar contrast patterns for splenic tissue: heterogeneous enhancement during arterial phases and homogeneous enhancement during venous phases. Additionally, testicular malignancies can present with a similar contrast pattern [4,9]. In discontinuous splenogonadal fusion, where the diagnosis may be more challenging, radiocolloid spleen scintigraphy (99mTc-sulphur colloid spleen-liver scan) may suggest the diagnosis. However, it is unlikely to preclude the need for surgical orchidectomy [4,10,11].

Preoperative diagnosis is challenging, particularly in discontinuous cases [1,4]. Furthermore, it is not a well-known disease, which means that the surgeon could not include it in the initial differential diagnosis of a testicular mass. The most feared consequence is unnecessary orchidectomy, since testicular cancer associated with splenogonadal fusion is extremely rare, with only five cases reported [5,12-15]. According to Carragher, up to 37% of splenogonadal fusion patients underwent unnecessary orchiectomy [16]. Thus, it is recommended the use of 99mTc-sulphur colloid spleen-liver scan and surgical exploration with extemporaneous examination to improve the diagnostic accuracy of this disease [5]. This approach enables appropriate treatment, including complete excision of splenic tissue while preserving the testicle [17].

## Conclusions

The diagnosis of splenogonadal fusion is rare and complex, requires several differential diagnoses, and is often made intraoperatively. The prognosis is excellent as long as there are no associated malformations. We intend to draw attention to the rarity of this diagnosis, to avoid an unnecessary orchidectomy.
